# Radical nephrectomy and regional lymph node dissection for locally advanced type 2 papillary renal cell carcinoma in an at-risk individual from a family with hereditary leiomyomatosis and renal cell cancer: a case report

**DOI:** 10.1186/s12885-016-2272-7

**Published:** 2016-03-17

**Authors:** Takao Kamai, Hideyuki Abe, Kyoko Arai, Satoshi Murakami, Setsu Sakamoto, Yasushi Kaji, Ken-Ichiro Yoshida

**Affiliations:** Department of Urology, Dokkyo Medical University, 880 Kitakobayashi Mibu, Tochigi, 321-0293 Japan; Division of Field Application, Life Technologies, Tokyo, Japan; PET Center, Dokkyo Medical University Hospital, Tochigi, Japan; Department of Radiology, Dokkyo Medical University, Tochigi, Japan

**Keywords:** Hereditary Leiomyomatosis and Renal Cell Cancer (HLRCC), Type 2 papillary renal cell carcinoma, Axitinib, Fumarate hydratase (*FH*), Targeted next-generation sequencing

## Abstract

**Background:**

Hereditary leiomyomatosis and renal cell carcinoma (HLRCC) is an autosomal dominant tumor susceptibility syndrome, and the disease-related gene has been identified as fumarate hydratase (fumarase, *FH*). HLRCC-associated kidney cancer is an aggressive tumor characterized by early metastasis to regional lymph nodes and distant organs. Since early diagnosis and provision of definitive therapy is thought to be the best way to reduce the tumor burden, it is widely accepted that germline testing and active surveillance for an at-risk individual from a family with HLRCC is very important. However, it still remains controversial how we should treat HLRCC-associated kidney cancer. We successfully treated the patient with locally advanced HLRCC-associated kidney cancer, who has received active surveillance because of at-risk individual, by radical nephrectomy and extended retroperitoneal lymph node dissection, and examined surgically resected samples from a molecular point of view.

**Case presentation:**

We recommended that 13 at-risk individuals from a family with HLRCC should receive active surveillance for early detection of renal cancer. A 48-year-old woman with a left renal tumor and involvement of multiple regional lymph nodes with high accumulation of fluorine-18-deoxyglucose on positron emission tomography was treated with axitinib as a neoadjuvant therapy. Preoperative axitinib induced the shrinkage of the tumor with decreased fluorine-18-deoxyglucose accumulation. Resected samples showed two thirds tumor tissue necrosis as well as high expression of serine/threonine kinase Akt and low expression of nuclear factor E2-related factor 2 (Nrf2) which activates anti-oxidant response and protects against oxidative stress in viable cancer cells. Targeted next-generation sequencing revealed that *FH* mutation and loss of the second allele were completely identical between blood and tumor samples, suggesting that *FH* mutation plays a direct role in *FH*-deficient RCC. She has remained well after radical operation for over 33 months.

**Conclusions:**

*FH* mutation plays a role in tumorigenic feature, a metabolic shift to aerobic glycolysis, and increased an anti-oxidant response phenotype in HLRCC-associated kidney cancer.

**Electronic supplementary material:**

The online version of this article (doi:10.1186/s12885-016-2272-7) contains supplementary material, which is available to authorized users.

## Background

Hereditary leiomyomatosis and renal cell cancer (HLRCC, Online Mendelian Inheritance in Man accession number 605839) is a recently identified autosomal dominant tumor susceptibility syndrome that is characterized by a predisposition to develop benign leiomyomas of the skin and the uterus (fibroids and myomas), as well as aggressive renal cell cancer with papillary type 2 (pRCC2) or collecting duct histology [1–3]. The disease-related gene has been identified as fumarate hydratase (fumarase, *FH*, Online Mendelian Inheritance in Man accession number 136850) located at 1q43. *FH* encodes an enzyme that is part of the mitochondrial tricarboxylic acid (TCA) cycle involved in cellular energy metabolism and appears to function as a tumor suppressor since its activity is very low or absent in tumors from individuals with HLRCC. HLRCC-associated kidney cancer has distinctive architectural and morphologic features, is particularly aggressive, and tends to metastasize to regional lymph nodes and distant organs early [4]. Therefore, a high detection rate of mutations in HLRCC families may enable early identification of at-risk individuals and allow early initiation of therapy while their tumors are still small. However, it stills remains controversial how we should treat HLRCC-associated kidney cancer [5]. So far, there have been several case reports regarding HLRCC-associated kidney cancer, however, most of those were reporting the mutation analysis of *FH*, pathological features, and clinical course. Furthermore, to our knowledge, there have been no case reports of the patients of at-risk of HLRCC-associated with kidney cancer who received active surveillance and were treated successfully, and little is known about the relationship between the clinicopathological features and molecular changes associated with targeting therapy in this disease. In the present study, we successfully treated a patient with locally advanced HLRCC-associated pRCC2 by neoadjuvant administration of axitinib and subsequent radical nephrectomy and extended retroperitoneal lymph node dissection.

*FH*-deficient RCC is characterized by enhanced aerobic glycolysis and increased anti-oxidant response phenotype [6, 7]. Overactivation of phosphatidylinositol 3‘kinase (PI3K), serine/threonine protein kinase B (Akt), and mammalian target of rapamycin (mTOR) pathway has been reported in RCC. Inhibition of Akt disrupts transcription of glucose transporter protein-1 (GLUT1) and its translocation to the plasma membrane to promote glucose utilization independent of an effect on cell proliferation [8]. Phosphorylation at two sites is required for full activation of Akt, since it is phosphorylated by PI3K-dependent kinase-1 (PDK1) at a threonine residue in the catalytic domain (Thr-308) and by PI3K-dependent kinase-2 (PDK2) at a serine residue (Ser-473) in the carboxy-terminal hydrophobic motif [9]. mTOR has dual rapamycin-sensitive (mTOR-raptor complex: mTORC1) and rapamycin-insensitive (mTOR-rictor complex: mTORC2) functions. mTORC1 is activated by PI3K-Akt and it phosphorylates S6 and eukaryotic translation initiation factor 4E-binding protein 1 (4EBP1), thereby promoting translation and protein synthesis. mTORC2 regulates the actin cytoskeleton and also possesses PDK2 activity that phosphorylates Ser-473 at the carboxy-terminus of Akt, which is essential for activation of Akt [10, 11], and mTORC2-pAkt(Ser-473) signaling affects energy metabolism and cell survival [12]. Activation of Akt may increase cell viability after inhibition of mTORC1 [9]. Hypoxia-inducible factor (HIF)1α expression is dependent on both raptor and rictor, whereas HIF2α expression only depends on rictor, with HIF2α and mTORC2 being more important in RCC [13]. Moreover, phosphorylation of Ser-473 in Akt is considered to be key molecular step in the progression of RCCs and could be a potential target [10, 11, 14]. Furthermore, available reports support HIF-dependent pseudo-hypoxia manner as the mechanism of tumorigenesis in HLRCC [15]. In *FH*-deficient kidney cancer cells, increased fumarate inactivate prolyl hydroxylases, leading to stabilization of HIF, and increased HIF target genes such as GLUT1, vascular endothelial growth factor (VEGF), platelet-derived growth factor (PDGF), and transforming growth factor (TGF)α, which facilitate tumor growth [7, 16].

On the other hand, HIF-independent manner has been recently reported [17]. *FH*-deficiency leads to succination of Kelch-like ECH-associated protein 1 (Keap1), stabilization of nuclear factor E2-related factor 2 (Nrf2), and induction of stress-response genes including HMOX1, which is important for the survival of *FH*-deficient cells. The Keap1-Nrf2 pathway is the major regulator of cytoprotective responses to oxidative and electrophilic stress. Although cell signaling pathways triggered by the transcription factor Nrf2 prevent cancer initiation and progression in normal and premalignant tissues, in fully malignant cells Nrf2 activity provides growth advantage by increasing cancer chemoresistance and enhancing tumor cell growth, and high Nrf2 protein level is associated with poor prognosis in cancer [18]. *FH* loss results in Keap1 inactivation and Nrf2-dependent activation of anti-oxidant pathways [19, 20].

Axitinib is a potent, selective, second-generation inhibitor of VEGF receptor (VEGFR) 1, 2, and 3 that blocks VEGFRs at sub-nanomolar drug concentrations [21], and relative potency of axitinib is 50–450 times greater than that of the first-generation VEGFR inhibitors like sorafenib or sunitinib [22]. In order to investigate the roles of Akt-mTOR pathway and Nrf2 anti-oxidant response element transcription pathway in HLRCC-associated kidney cancer, we examined the expressions of phosphorylated-Akt (Ser-473) (pAkt(Ser-473), phosphorylated-Akt (Thr-308) (pAkt(Thr-308), phosphorylated-S6 ribosomal protein (Ser-235/236) (pS6), and Nrf2 in surgically resected samples. We also investigated *FH* mutations by sequencing the coding exons and intron flanking regions in both blood and tumor samples by targeted next-generation sequencing analysis. Such information might be useful to understand the signaling pathway in HLRCC-associated kidney cancer from a molecular point of view.

## Case presentation

A 48-year-old woman (III-8, a sister of the proband from this HLRCC family) underwent abdominal ultrasonography annually at a local clinic after 2007, and presented with a left renal mass detected by an ultrasonography and was introduced to our hospital in March 2013 (Additional file 2: Figure S1).

**Fig. 1:**
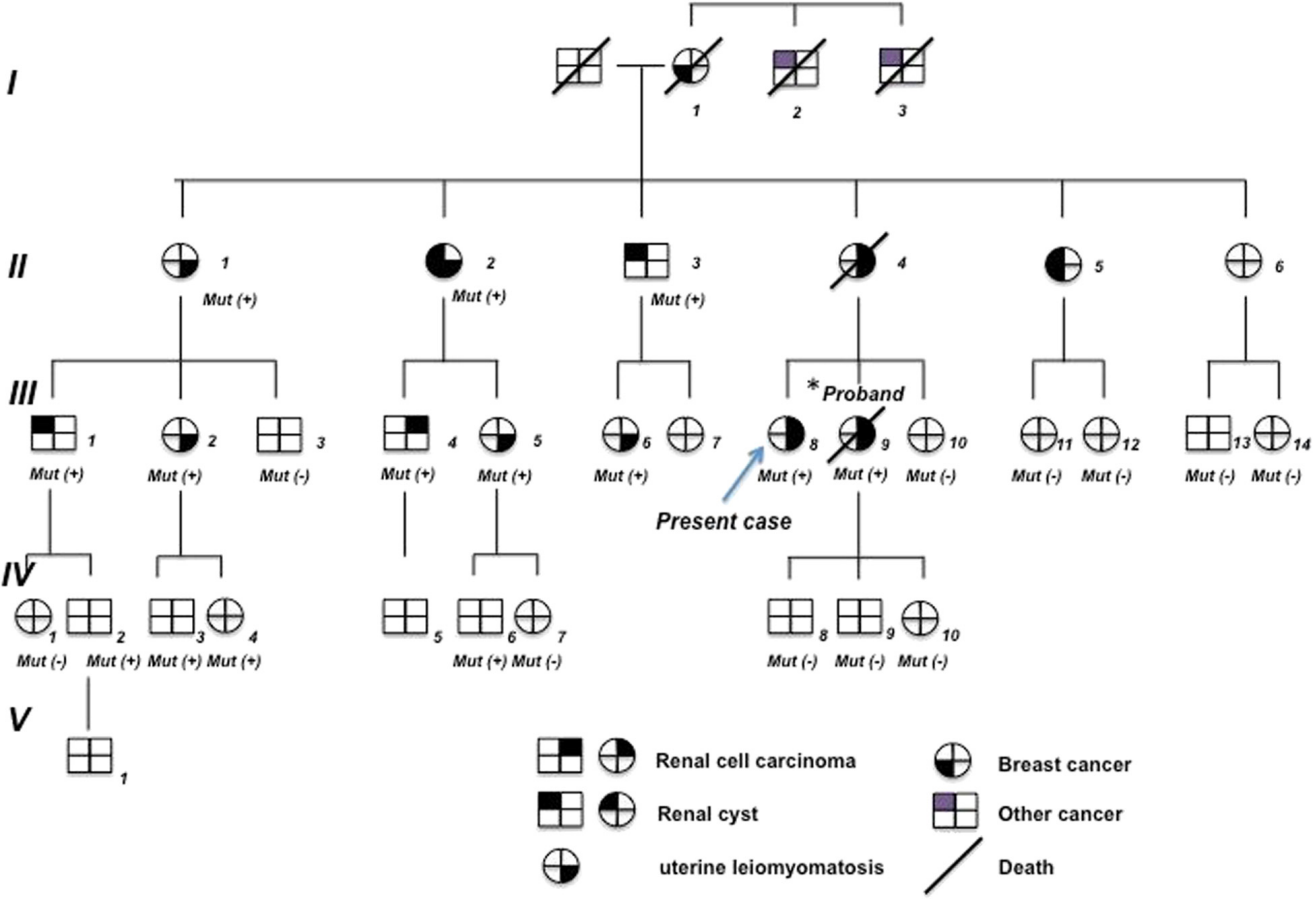
Pedigree. Generations are represented by Roman numerals and individuals are shown by Arabic numerals. The present patient is III-8 (indicated by the arrow) and the proband is III-9. “Mut” shows mutation screening. “Mut +” and “Mut −” indicate mutation-positive and mutation-negative individuals, respectively

She had undergone enucleation myomectomy for uterine leiomyomatosis at the age of 29 years at another hospital, while hysterectomy had been performed for recurrence large uterine leiomyomatosis at the age of 39 years at other hospital. In 2007 (when she was 40), her sister was diagnosed with HLRCC having a novel *FH* mutation at 241,671,938 bp (C574T) by direct sequencing of the *FH* gene from leukocyte DNA. Her sister subsequently died of HLRC-associated advanced renal cancer. In 2007, sequencing of DNA extracted from blood cells of this patient confirmed that she also had the same *FH* mutation as her sister [23]. After 2007, we recommended that 13 members of this family with the *FH* mutation should receive active surveillance by annual imaging (abdominal plain computed tomography (CT) or ultrasonography) at a convenient clinic (Fig. 1).

**Fig. 2:**
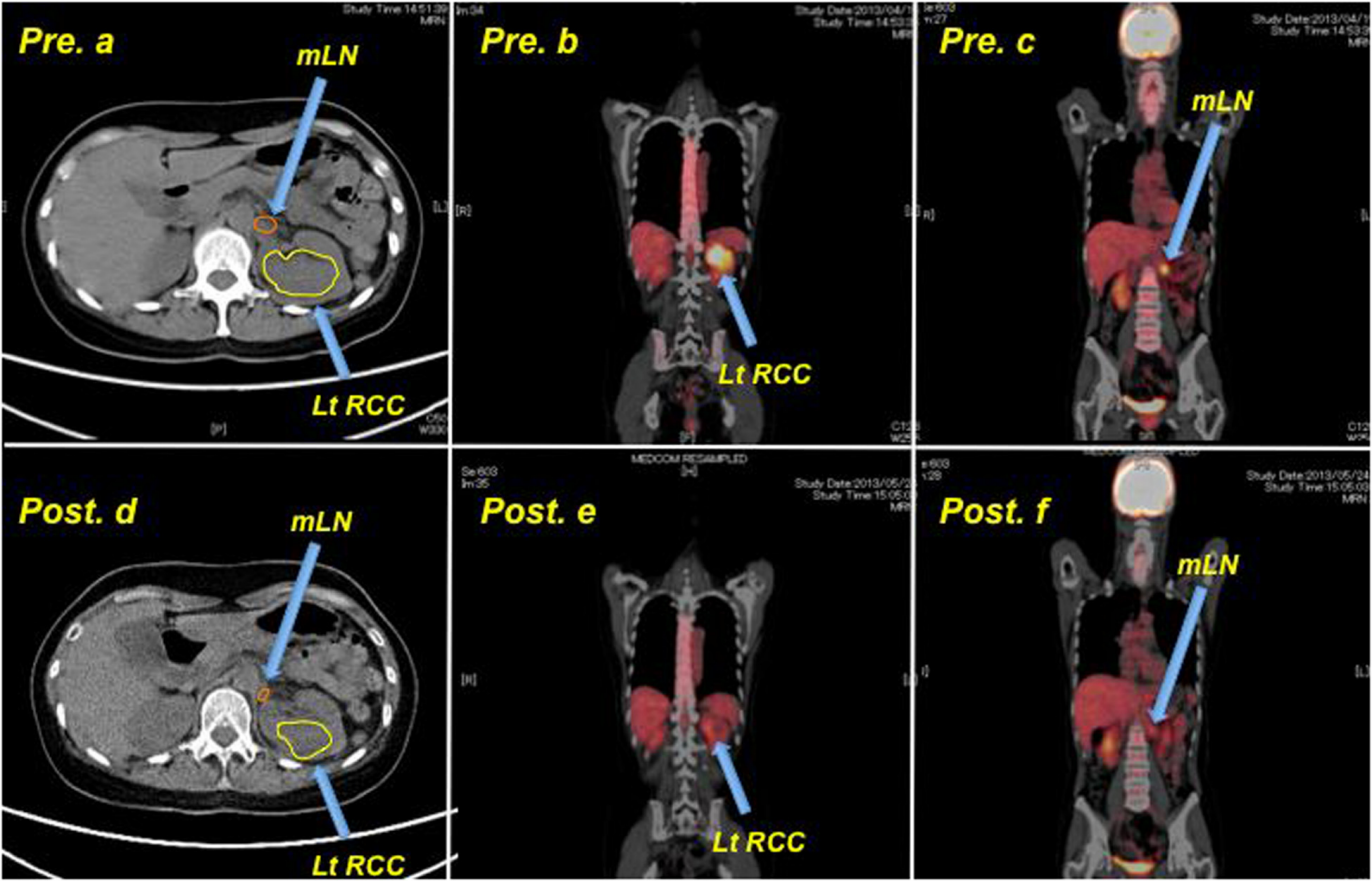
Positron emission tomography (PET) with [18 F] fluorodeoxyglucose (18 F-FDG PET) / plain computed tomography (CT). Pre: Before treatment with axitinib. Post: After administration of axitinib for 4 weeks. **a**, **d**: Plain abdominal CT shows that the primary left renal tumor and enlarged lymph nodes have decreased in size. **b**, **e**: SUVmax of the primary tumor decreased from 15.3 to 2.9 after administration of axitinib. **c**, **f**: SUVmax of the regional lymph nodes decreased from 7.5 to 2.3 after administration of axitinib

Laboratory tests revealed moderate anemia (hemoglobin: 9.3 g/dl) and elevation of serum C-reactive protein (CRP: 3.19, normal < 0.3 mg/dl). Karnofsky performance status (KPS) was 100 %. Plain CT scans obtained at our hospital showed a left renal tumor with a diameter of 7 cm and involvement of multiple regional para-aortic lymph nodes, but no distant metastases (cT3aN1M0) (Fig. 2a). Positron emission tomography (PET) showed fluorine-18-deoxyglucose (FDG) accumulation in the renal tumor and the metastatic lymph node and the maximum standardized uptake value (SUVmax) was 15.3 and 7.5, respectively (Figs. 2b,c).

Her risk classification for renal cancer was intermediate risk according to the Memorial Sloan-Kettering Cancer Center (MSKCC) criteria. However, the prognosis of patients with HLRCC-associated renal cancer, in particular those with extrarenal involvement, is extremely poor. Furthermore, her tumors showed a different imaging pattern from that of typical clear cell RCC (Additional file 2: Figure S1), and the histology of the renal cancers in her relatives was non-clear cell RCC (undifferentiated RCC in her mother, pRCC2 in both her sister and maternal cousin). Thus, the tumor of this patient seemed likely to be non-clear cell carcinoma, but we did not perform needle biopsy to avoid dissemination of cancer cells.

In order to decrease the tumor burden and improve the feasibility of surgery, we selected preoperative treatment with a multi-targeted tyrosine kinase inhibitor (TKI). In comparison to first-generation TKIs targeting the VEGFR, axitinib is a potent second-generation inhibitor of VEGFRs with a higher affinity for tyrosine kinase and achieves stronger inhibition of kinase activity with fewer adverse effects such as thrombocytopenia. Additionally, first-generation inhibitors block other targets, such as PDGF receptors (PDGFR), KIT (cluster of differentiation 117: CD117), b-rapidly accelerated fibrosarcoma (RAF), and Fms-like tyrosine kinase 3 (FLT-3), which are not substantially inhibited by axitinib. These off-target activities might contribute to the adverse effects of the first-generation inhibitors, suggesting that more specific inhibitors of VEGFR such as axitinib might have an enhanced therapeutic window. We recently successfully treated a patient who had a large right RCC showing sarcomatoid differentiation that directly invaded the duodenum and inferior vena cava with regional lymph node involvement. In this patient, radical right nephrectomy, cavotomy with thrombectomy, and pancreatoduodenectomy were successfully performed after administration of axitinib as first-line neoadjuvant therapy without severe toxicity [24].

We selected axitinib as preoperative molecular-targeting therapy to decrease the tumor size before surgery with good tolerability. Administration of axitinib starting at 5 mg/day was scheduled for four to six weeks before radical surgery involving left nephrectomy and extended retroperitoneal lymph node dissection (para-aortic and aorto-caval nodes). After 1 week, the dose of axitinib was increased to 14 mg/day. After four weeks of total dose of axitinib of 329 mg (5 mg/day for continuous 7 days and 14 mg/day for following continuous 21 days), there were no apparent adverse events of > grade 3, excluding headache and hypertension (systolic blood pressure > 200 mmHg). Tumor shrinkage and a decrease of SUVmax were observed (Figs. 2d-f). Subsequently, we successfully carried out radical left nephrectomy and extended retroperitoneal lymph node dissection (para-aortic and aorto-caval nodes). Macroscopically, the tumor was an invasive whitish-yellowish mass with partial necrosis. Pathological examination confirmed pRCC2 with Fuhrman grade 3 differentiation (pT3apN1M0). The pathological effect of axitinib was grade 2 (i.e., two-thirds necrosis of the tumor). The patient has been receiving axitinib at 5 mg/day in the manner of one cycle of one week (5 days on - 2 days off) as adjuvant therapy for 33 months, and remains well with no evidence of recurrence at 33 months after the operation.

## Materials and methods

### Western blotting and Immunohistochemistry

We performed Western blotting using a rabbit anti-human antibody targeting pAkt (Ser-473) (Cell Signaling Technology, Inc; PhosphoPlus Akt (Ser-473) Antibody Kit; # 9270, Danvers, MA), a rabbit anti-human antibody for pAkt (Thr-308) (Cell Signaling Technology, Inc; Phospho-Akt (Thr308) Antibody Kit; # 2965, Danvers, MA), a rabbit anti-human antibody targeting phosphorylated ribosomal protein S6 kinase (pS6) (2 F9, Cell Signaling Technology, Inc; # 4856), as described previously [25].

Immunohistochemical staining was performed with anti-Nrf2 monoclonal antibody (abcam, # ab-62352, Cambrige, UK) using the immunoperoxidase technique and microwave treatment of tissue sections in citrate buffer as described previously [26].

For comparison to the present case, we examined the expression of pAkt (Ser-473), pAkt (Thr-308) and pS6 in surgical specimens of five patients with locally advanced clear cell RCCs with pT3bpN1 or pT4 who received preoperative axitinib as well as the current patient, and of Nrf2 in HLRCC-associated kidney cancer tissues of the proband (III-9) and maternal cousin (III-4) who received no prior treatment.

### DNA samples

Germline DNA was extracted from leukocytes according to the standard protocols. Frozen tumor samples were ground to a powder in liquid nitrogen and 30–50 mg of the sample was used for DNA extraction with the AllPrep kit (Qiagen). DNA was quantified and its purity assessed with a NanoDrop ND-1000 spectrophotometer (Labtech).

### Next-generation sequencing

We investigated *FH* mutations by sequencing the coding exons and intron flanking regions in both blood and tumor samples. For targeted next-generation sequencing analysis, the custom primers for *FH* region were designed using Ampliseq Designer (Life Technologies). Library construction and sequencing were carried out using Ion AmpliSeq Library Kit 2.0, Ion PGM IC 200 kit and Ion PGM (Life Technologies) according to the manufacturer's instructions (Additional file 1).

### Data analysis

After a sequencing reaction, the raw signal data were analyzed using Torrent Suite version 4.2.1. The pipeline includes signaling processing, base calling, quality score assignment, adapter trimming, mapping to GRCH37/hg19 reference, detection of mapping quality, and variant calling. After completion of the primary data analysis, a list of detected alleles, sequence variant [single-nucleotide Polymorphisms (SNPs) and the insertion or the deletion (Indels)] were compiled in a variant call file format and presented via the web-based user interface. The results of mapping and variant calling were visualized using Integrative Genome viewer (Broad Institute) (Additional file 1).

## Results

### Expression of pAkt and pS6

**Fig. 3:**
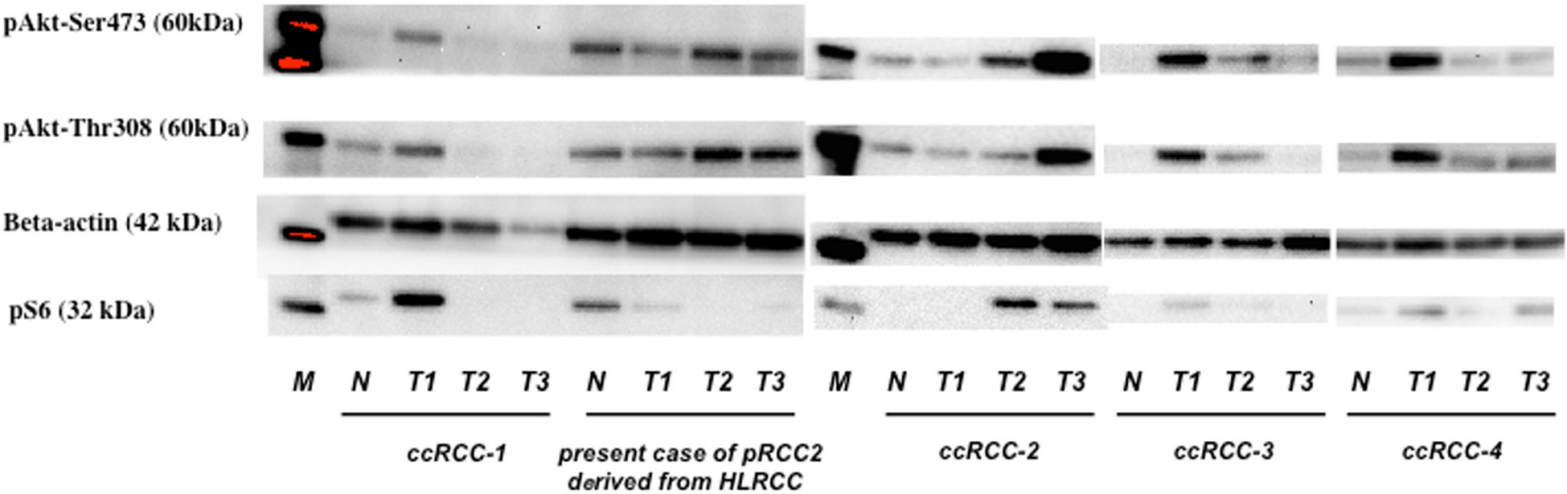
Western blotting. Western blotting for surgically resected tissues (M: marker, N: normal tissue, T1-3: three different parts of tumor tissues). In a patient with cT3bN1M1 clear cell renal cell carcinoma (ccRCC-1) who received preoperative axitinib as well as the current patient, tumor tissues obtained by nephrectomy after axitinib treatment showed heterogeneous changes. Some tumor tissue (T2 and T3) showed much lower expression of pAkt (Ser-473), pAkt (Thr-308), and pS6 than other tissue (T1). Similarly, in the other patients with cT_any_N1M_any_ ccRCCs (ccRCC-2 to −4) treated with preoperative axitinib, tumor tissues showed heterogeneous pattern. These findings indicate that some parts of the cancer would show a good response to axitinib but other parts would not. On the other hand, in the present patient with cT3aN1M0, renal cell cancer with papillary type 2 (pRCC2), surgically resected cancer tissues (T1 to T3) showed high expression of pAkt (Ser-473) and pAkt (Thr-308), as well as very low expression of pS6, indicating that the mTORC2-Akt signaling may be more important for molecular targeting than the mTORC1-S6 pathway in HLRCC-associated kidney cancer compared with clear cell RCC

In six patients, five clear cell RCCs and this case, receiving preoperative axitinib treatment, similar findings were observed (shrinkage of the tumor, decreased SUVmax of the tumor, and two thirds tumor tissue necrosis). For the other patients with cT3bN1 or cT4 clear cell RCC, tumor tissues showed heterogeneous changes. Some of tumor tissues showed much lower expression of pAkt (Ser-473), pAkt (Thr-308), and pS6 than other tissues. On the other hand, in our current patient with cT3aN1M0, pRCC2, tumor tissues showed high expression of pAkt (Ser-473) and pAkt (Thr-308), as well as very low expression of pS6 (Fig. 3).

### Expression of Nrf2

**Fig. 4:**
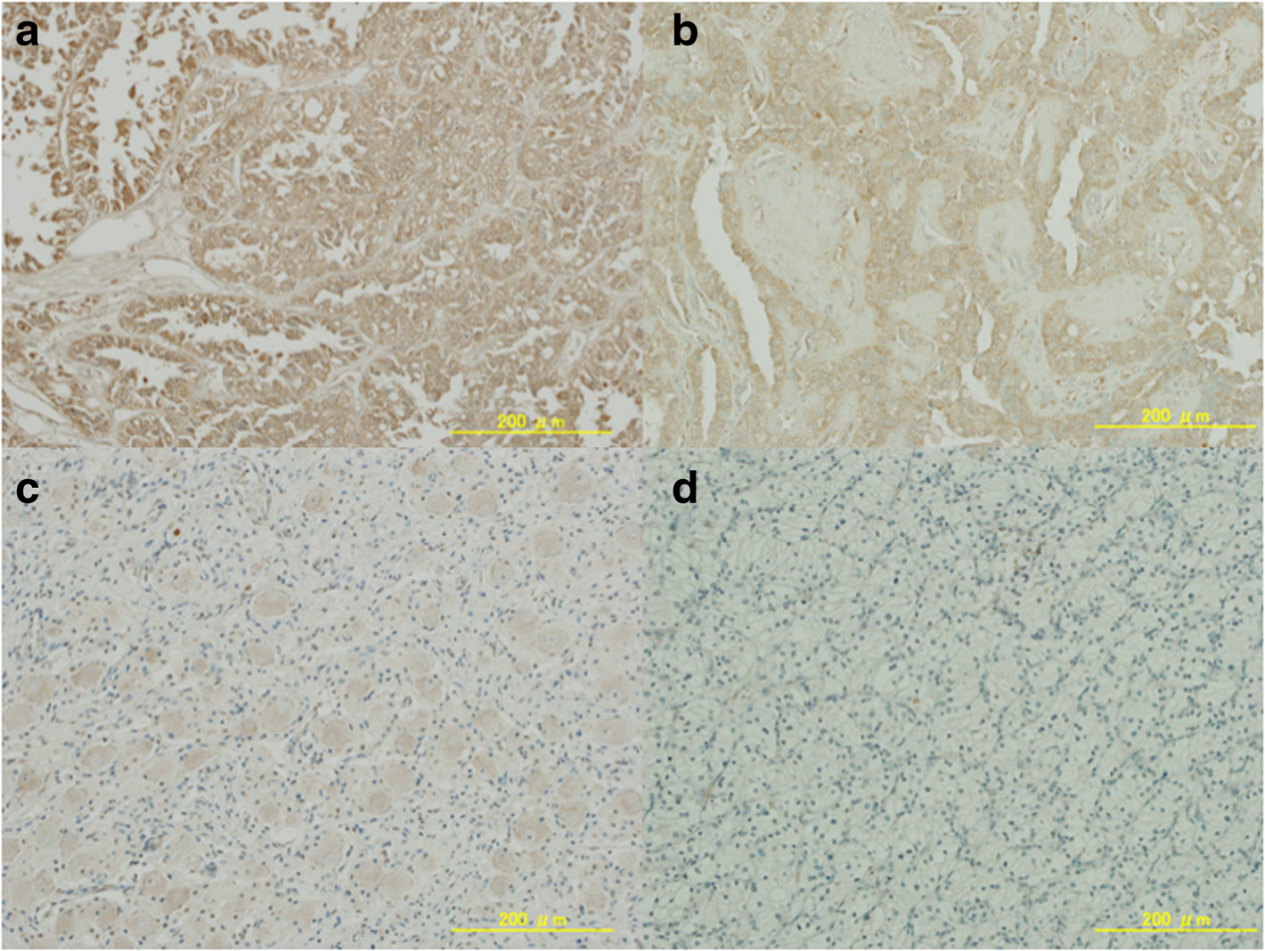
Immunohistochemistry. Immunohistochemical analysis of Nrf2 expression using anti-Nrf2 monoclonal antibody in HLRCC-associated kidney cancer (a-c) (X 200 magnification. Scale bars showed 200 μm). **a**, **b**: Much of cancer cells showed diffuse strong brown staining in a membrane and cytoplasm for anti-Nrf2 antibody in the proband (III-9) (**a**) and maternal cousin (III-4) (**b**) with non-prior therapy. **c**: Some of viable cancer cell showed weak reaction in the present case (III-8) with neoadjuvant axitinib therapy. **d**: Clear cell renal cell carcinoma tissues randomly selected for this study showed negative staining

While much of tumor cells showed diffusely strong reaction for anti-Nrf2 antibody in the proband (III-9) and maternal cousin (III-4) (Figs. 4a, 4b), some of viable tumor cells showed weak staining in the present case (III-8) (Fig. 4c). Normal kidney and clear cell RCC tissues showed negative staining (Fig. 4d).

### Molecular genetic analysis

The average Ion PGM™ sequencing output per sample was 150 mega bases with 0.9 million sequencing reads. Of the 16 amplicons in the FH-gene, 100 % achieved a minimum average sequencing depth of 500X and mean depth were 28,419X-34,591X. In samples, the Ion PGM™ detected SNPs and deletions, details of results are shown in Table 1. In the blood and resected kidney cancer tissue samples from this patient and the proband (III-9), common SNPs on exon5 (position; 241,671,938 bp, C574T, codon p. H235Y) was detected, and new alleles were detected at intron regions.

**Table 1:** Allele detection of *FH*-gene using next-generation DNA sequencer

Sample	Chrom	Position	Ref	Variant	Allele Call	Frequency	Quality	Type	Coverage
Blood	chr1	241667244	G	T	Homozygous	100	3825.7	SNP	399
	chr1	241669249	C	T	Homozygous	100	3797.23	SNP	398
	chr1	241671938	G	A	Heterozygous	63.5	1405.16	SNP	400
	chr1	241675240	A	–	Homozygous	100	2791.44	DEL	387
	chr1	241682820	G	T	Homozygous	100	3806.12	SNP	397
Cancer tissue	chr1	241667244	G	T	Homozygous	100	3853.67	SNP	400
	chr1	241669249	C	T	Homozygous	100	3853.67	SNP	400
	chr1	241671938	G	A	Heterozygous	45	669.28	SNP	400
	chr1	241675240	A	–	Homozygous	100	2733.7	DEL	383
	chr1	241682820	G	T	Homozygous	100	3796.5	SNP	396
Positive Ctrl^a^	chr1	241667233	C	T	Heterozygous	55.5	1058.17	SNP	400
	chr1	241667244	G	T	Heterozygous	51.3	888.08	SNP	398
	chr1	241675240	A	–	Homozygous	100	2622.8	DEL	385

Positive Ctrl^a^ : Centre d'Étude du Polymorphisme Human, http://www.cephb.fr/ (for chromosome 2 linkage map and DNA from individual 1347–02)

The average Ion PGM™ sequencing output per sample was 150 mega bases with 0.9 million sequencing reads. Of the 16 amplicons in the FH-gene, 100 % achieved a minimum average sequencing depth of 500X and mean depth were 28,419X-34,591X. In samples, the Ion PGM™ detected single-nucleotide Polymorphisms (SNPs) and deletions, details of results are shown in Table 1. In blood sample and cancer tissue sample of the current case, SNPs same as the past report [7], hetero on FH-gene exon5, was detected, and new alleles were detected at intron regions

SNPs of chr1:241,667,244 bp and chr1:241,675,240 were common between blood, cancer samples and normal human cell (CEPH individuals 1347–02 control DNA, Lifetechnologies). This result indicates these SNPs have low association with cancer. Other variants were located at intron; such mutations may cause a proportion of mature messenger RNA with improperly spliced intron sequences. So we will try gene expression profiling, RNA-seq, for these samples including fusion-gene analysis

## Discussion and conclusions

In the present study, next-generation sequencing revealed that *FH* mutation and loss of the second allele were completely identical between blood and tissue samples from this patient and her sister (III-9) who died of advanced HLRCC-associated kidney cancer, indicating that *FH*-deficient RCC is a unique neoplasm that progresses directly by *FH* mutation.

In *FH*-deficient RCC, oxidative phosphorylation is impaired and the cells undergo a shift to aerobic glycolysis, consistent with the Warburg effect [6, 7]. The conversion of glucose metabolism from oxidation to glycolysis, the Warburg effect, is one of the representative strategies for generation of adenosine triphosphate (ATP) in cancer cells [27]. Reprogramming of energy metabolism, the conversion of glucose metabolism from oxidation to glycolysis, the Warburg effect, can now be viewed as one of the “hallmarks of cancer” [28]. RCC is characterized by impaired oxidative phosphorylation and a metabolic shift to aerobic glycolysis, a form of metabolic reprogramming. In particular, HLRCC-associated kidney cancer cells have lost the ability to completely cycle through the TCA cycle, due to the loss of *FH* enzyme activity, and have effectively lost the ability to perform oxidative phosphorylation indicating that these cancers exist in a state of enforced dependence upon glycolysis and represent a notable example of the Warburg effect [7]. Thus, HLRCC-associated kidney cancer might be a clinical model to study energy metabolism deregulation, as well as developing new targeted therapeutic approaches for TCA cycle enzyme-deficient cancers [29]. In this HLRCC-associated pRCC2 case, surgically resected cancer tissue showed high expression of pAkt (Ser-473) and pAkt (Thr-308), as well as very low expression of pS6, indicating that we should study the roles of mTORC2-Akt signaling in HLRCC-associated kidney cancer from the metabolic point of view in more patients in the forthcoming study.

On the other hand, *FH*-deficient RCC is also characterized by increased oxidative stress and elevated levels of reactive oxygen, thus effective anti-oxidant response is critical for continued growth [6, 7]. In this study, subsequent immunohistochemical staining for Nrf2 protein in the HLRCC-associated pRCC2 also showed intense positive staining (III-9 and −4). At the same time, normal kidney and clear cell RCC tissues showed negative staining. Our findings were consistent with those by previous study [20], indicating that Nrf2 was indeed activated in these *FH*-deficient RCC tissues. Thus, *FH*-deficient RCC appeared to be linked to increased expression of anti-oxidant genes with accompanied by the accumulation of Nrf2. In this HLRCC-associated pRCC2 case treated with neoadjuvant axitinib, some of viable cancer cells showed weak reaction for anti-Nrf2 antibodies compared to the tumors of the proband (III-9) and maternal cousin (III-4) who received no prior treatment in which much of cancer cells showed diffusely strong reaction, indicating that axitinib might suppress the Nrf2 pathway by unknown mechanism. Since we do not have preoperative tumor tissues and stored blood samples, we could not compare the VEGF levels between before and after administrating axitinib in the present study. However, in addition to pAkt, pS6, and Nrf2, we should also analyze repeatedly the VEGF, GLUT1 or HIF using tissue and blood samples in order to correspond to dynamic change of the tumor and the general condition which is continuously changed with time in the future.

From a molecular point of view, insight in the cellular pathways involved in pathogenesis of HLRCC might lead to specific options for early diagnosis and targeted therapies. However, since this is only a single case report about our experience with surgery after axitinib treatment for HLRCC-associated kidney cancer, the results should be interpreted with consideration of such limitations and definite conclusions cannot be obtained. While an investigation of the usefulness of axitinib for preoperative or neoadjuvant therapy in patients with locally advanced RCC is now ongoing, availability of axitinib for adjuvant therapy for RCCs has not yet elucidated. Although there was no detailed information regarding histological type of papillary RCC in a previous study using other kinase inhibitors in papillary RCCs, sunitinib seems to be more effective than sorafenib [30]. Furthermore, a phase II trial of bevacizumab and erlotinib in patients with advanced HLRCC-associated pRCC2 as well as sporadic pRCC2 is currently under way (NCT01130519). Therefore, it would be great to conduct in vitro studies using established two HLRCC kidney cancer lines, UOK262 and UOK268 [29, 31].

It is thought that early diagnosis and provision of definitive therapy is the best way to reduce the tumor burden as rapidly as possible. Unlike other hereditary renal cancers, HLRCC-associated kidney cancer is an aggressive tumor characterized by metastasis to regional and distant lymph nodes [4]. A recent report regarding HLRCC-associated kidney cancer recommended that surveillance should preferably be annual abdominal MRI, and that treatment of renal tumors should be prompt and generally involve wide surgical excision with consideration of retroperitoneal lymph node dissection [5]. Thirteen at-risk individuals from this HLRCC family had received active surveillance by annual imaging (abdominal plain CT or ultrasonography) at a convenient clinic, however, as shown in Fig. 2 and Additional file 2: Figure S1, we may overlook the smaller tumors associated with HLRCC in plain CT and/or ultrasonography because of low contrast of the tumors to the normal kidney. Although enhanced CT may be useful, given the radiation exposure and the adverse effect of contrast medium, magnetic resonance imaging (MRI) seems to be suitable for surveillance as recommended [5]. Subsequently, we would ask their attending physicians at a convenient clinic annual MRI imaging study every twelve months.

Currently, surgical intervention is the only therapy available to patients with HLRCC­associated kidney cancers. The patient underwent radical left nephrectomy and extended retroperitoneal lymph node dissection (para-aortic and aorto-caval nodes) after administration of preoperative axitinib. It is difficult issue how we should follow the patient. The patient undergoes imaging examination of chest CT and abdominal MRI at least every three months and PET scan every six months, and remains well with no evidence of recurrence at 33 months after the operation. The patient is receiving axitinib as adjuvant therapy, and we would like to decrease the dose of axitinib gradually. Careful patient selection and meticulous surgical technique are essential in the treatment of patients with HLRCC-associated kidney cancer, and these points should be further emphasized in the era of targeted therapy. Collection of HLRCC-affected family data dedicated to monitoring of patients will provide information on clinical variability and outcome measures that will allow clinicians to adjust diagnostic criteria and management recommendations. It is our hope that more patients with HLRCC-associated kidney cancer will be able to achieve a better outcome.

## Ethics approval

The patient and many of individuals in this family signed a consent form that was approved by our institutional Committee on Human Rights in Research for the analysis of germline and somatic DNA. On the other hand, some individuals could not come to our hospital and we could not get approval for DNA analysis, however, all participants in this family give approval for the publication of their clinical and other details in written informed consent. Furthermore, if the participant has died, then consent for publication has been sought from the next of kin of the participant. Thus, we have 'consent to publish' from all the individuals represented in the family tree in Fig. 1. This study was conducted in accordance with the Helsinki Declaration and was approved by the Dokkyo Medical University Hospital ethical review board.

## Consent

Written informed consent was obtained from the patient and their relatives to sequence their DNA and for the publication of this case report and any accompanying sequence data or images. A copy of the written consent is available for review by the Editor of this journal.

## Additional files

Additional file 1: Supplementary Materials and Methods. (DOCX 270 kb)

Additional file 2: Figure S1. Imaging of the left renal tumor. a: Ultrasonography, b: Enhanced CT, c: MRI T2W1, d: MRI gadolinium. Lt RCC: left renal cell carcinoma. mLM: metastatic lymph node tumor. These imaging were at pre-treatment with axitinib. (TIFF 1162 kb)

## Abbreviations

4EBP1: 4E-binding protein 1
Akt: protein kinase B
ATP: adenosine triphosphate
ccRCC: clear cell renal cell carcinoma
Chrom: chromosome
CRP: C-reactive protein
CT: computed tomography
DNA: deoxyribonucleic acid
FDG: fluorine-18-deoxyglucose
FH: fumarate hydratase
FLT-3: Fms-like tyrosine kinase 3
GLUT1: glucose transporter protein-1
HIF: hypoxia-inducible factor
HLRCC: Hereditary Leiomyomatosis and Renal Cell Cancer
Indels: the insertion or the deletion
Keap1: Kelch-like ECH-associated protein 1
KIT: cluster of differentiation (CD)117
KPS: Karnofsky performance status
MRI: magnetic resonance imaging
MSKCC: Memorial Sloan-Kettering Cancer Center
mTOR: mammalian target of rapamycin
mTORC1: mTOR-raptor complex
mTORC2: mTOR-rictor complex
Mut: mutation
Nrf2: nuclear factor E2-related factor 2
pAkt: phosphorylated-Akt
PCR: polymerase chain reaction
PDGF: platelet-derived growth factor
PDGFR: PDGF receptor
PDK1: PI3K-dependent kinase-1
PDK2: PI3K-dependent kinase-2
PET: positron emission tomography
PI3K: phosphatidylinositol 3‘kinase
pRCC2: renal cell cancer with papillary type 2
pS6: phosphorylated-S6 ribosomal protein
RAF: rapidly accelerated fibrosarcoma
RCC: renal cell carcinoma: Ref, references
Ser: serine
RNA: ribonucleic acid
SNPs: single-nucleotide polymorphisms
SUVmax: maximum standardized uptake value
TCA: tricarboxylic acid
TGF: transforming growth factor
Thr: threonine
TKI: tyrosine kinase inhibitor
VEGF: vascular endothelial growth factor
VEGFR: VEGF receptor
