# Supplementary material for: Radical nephrectomy and regional lymph node dissection for locally advanced type 2 papillary renal cell carcinoma in an at-risk individual from a family with hereditary leiomyomatosis and renal cell cancer: a case report
Source: BMC Cancer. 2016 Mar 17;16:232. doi: 10.1186/s12885-016-2272-7 (PMC4794818; doi:10.1186/s12885-016-2272-7)
Supplement: Supplementary file 1 — Supplementary Materials and Methods. (DOCX 270 kb) [file 12885_2016_2272_MOESM1_ESM.docx]

**Next-generation sequencing**

For targeted next-generation sequencing analysis, the custom primers were designed using Ampliseq™ Designer (Life Technologies), design tool to generate two pools of 16 primers for amplification of genomic regions of interest. Primers were designed for covering 10 FH exons(1,638bp, Fig.1). It was used for amplification of isolated DNA in a multiplex PCR. In more details, 10 ng of DNA was amplified by PCR using the premixed primer pool and Ion AmpliSeq HiFi master mix (Ion AmpliSeq Library Kit 2.0). For library construction, the resulting 16 multiplexed amplicons were treated with a FuPa reagent to partially digest primer sequences and phosphorylate the amplicons. The amplicons were then ligated to adapters with the addition of barcodes from the Ion Xpress Barcode Adapters 1–16 kit according to the manufacturer's instructions (Life Technologies). After ligation, the amplicons underwent nick translation and additional library amplification by PCR to complete the linkage between adapters and amplicons. Library concentration and amplicon size were confirmed using an Agilent BioAnalyzer high sensitivity DNA kit (Agilent Technologies). Next, multiplexed barcoded libraries were enriched by clonal amplification using emulsion PCR on Ion Sphere particles (Ion PGM™ IC 200 kit) and loaded on an Ion 318 Chip using Ion Chef. Massively parallel sequencing was carried out on Ion PGM™ (Lifetechnologies) using the on PGM™ IC 200 kit according to the manufacturer's instructions.

### Data analysis

After a sequencing reaction, the raw signal data were analyzed using Torrent Suite version 4.2.1. The pipeline includes signaling processing, base calling, quality score assignment, adapter trimming, mapping to GRCH37/hg19 reference, detection of mapping quality, and variant calling. After completion of the primary data analysis, a list of detected alleles, sequence variant [SNPs and Indels] were compiled in a variant call file format and presented via the web-based user interface. The results of mapping and variant calling were visualized using Integrative Genome viewer (Broad Institute).


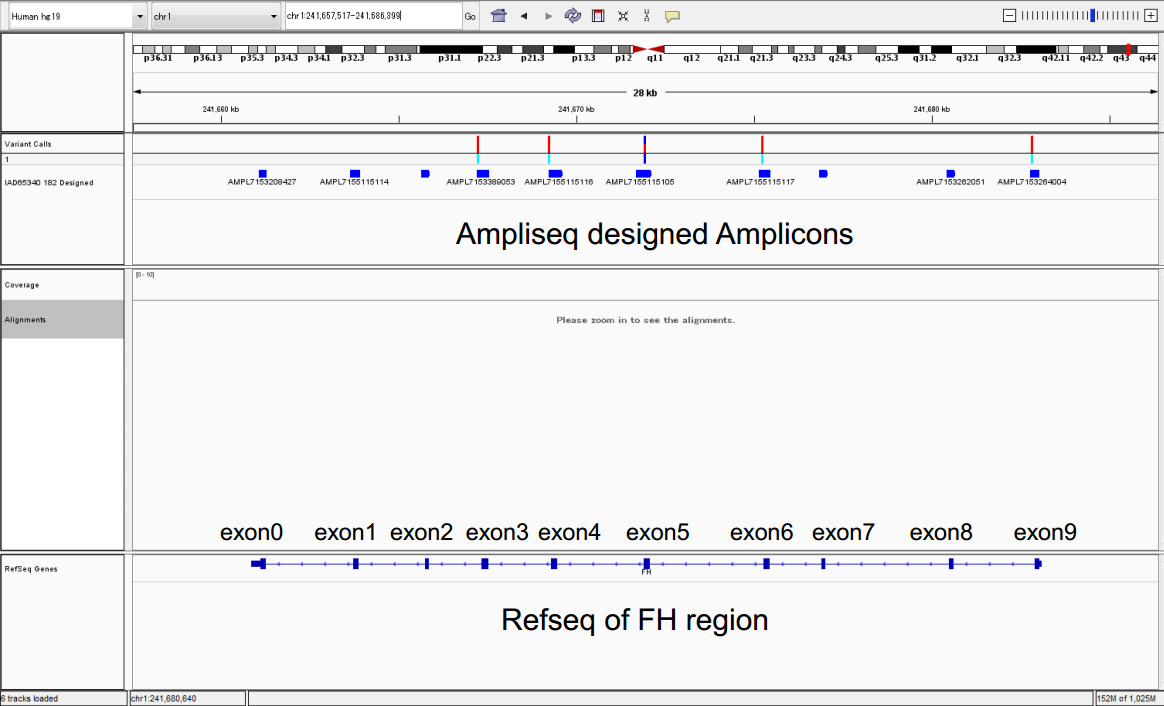


Supplementary Figure.1. Summary of primer design for FH regions.

All of FH regions, 10 exons (1,638bp) were covered with 16 amplicons, amplicons size were approximately 275bp.

**Results**

The average Ion PGM™ sequencing output per sample was 150 mega bases with 0.9 million sequencing reads. Of the 16 amplicons in the FH-gene, 100% achieved a minimum average sequencing depth of 500X and mean depth were 28,419X-34,591X. In two samples, the Ion PGM™ detected single-nucleotide Polymorphisms (SNPs) and deletions.


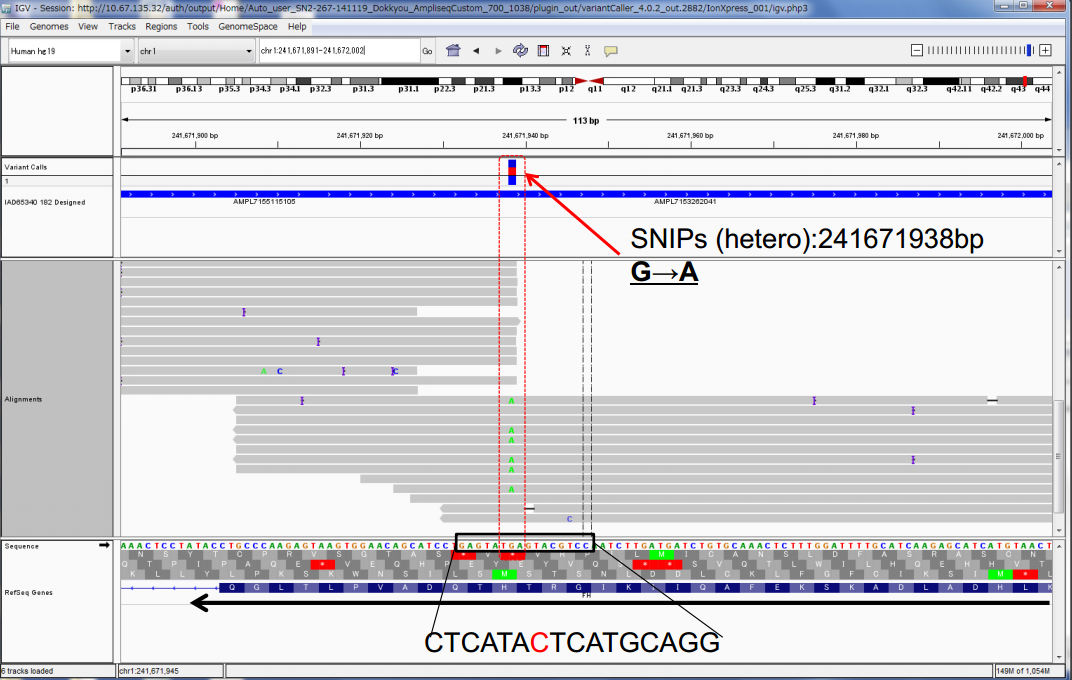


Supplementary Figure. 2. Common SNPs with the past report.

In blood and tissue samples, common SNPs on exon5 (position; 241,671,938bp, C574T, codon p. H235Y) was detected.
