# Supplementary figures and images for: Radical nephrectomy and regional lymph node dissection for locally advanced type 2 papillary renal cell carcinoma in an at-risk individual from a family with hereditary leiomyomatosis and renal cell cancer: a case report
Source: BMC Cancer. 2016 Mar 17;16:232. doi: 10.1186/s12885-016-2272-7 (PMC4794818; doi:10.1186/s12885-016-2272-7)

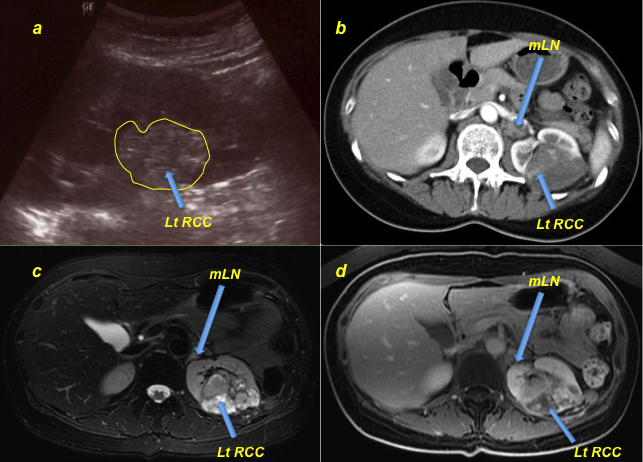

Supplement: Supplementary file 2 — Imaging of the left renal tumor. a: Ultrasonography, b: Enhanced CT, c: MRI T2W1, d: MRI gadolinium. Lt RCC: left renal cell carcinoma. mLM: metastatic lymph node tumor. These imaging were at pre-treatment with axitinib. (TIFF 1162 kb) [file 12885_2016_2272_MOESM2_ESM.tiff]
